# A Rare Case of Staphylococcus caprae-Caused Periprosthetic Joint Infection Following Total Hip Arthroplasty: A Literature Review and Antibiotic Treatment Algorithm Suggestion

**DOI:** 10.7759/cureus.39471

**Published:** 2023-05-25

**Authors:** Philip Domashenko, Georgios Foukarakis, Eustathios Kenanidis, Eleftherios Tsiridis

**Affiliations:** 1 Department of Orthopedics, Tsiridis Orthopedic Institute - ICAROS Clinic, Thessaloniki, GRC; 2 Department of Orthopedics, Centre of Orthopedic and Regenerative Medicine Research (CORE) Center for Interdisciplinary Research and Innovation (CIRI) Aristotle University of Thessaloniki, Thessaloniki, GRC

**Keywords:** orthopedic implant-related infection, treatment choices, total hip arthroplasty, periprosthetic joint infection, staphylococcus caprae

## Abstract

In this study, we discuss a case of a 59-year-old male who developed a periprosthetic joint infection (PJI) three months after a total hip arthroplasty (THA). The patient complained of groin and buttock pain, swelling, and high temperature. A palpable fluid collection, discomfort, edema, and elevated local temperature were present in the clinical examination. Laboratory analysis revealed elevated white blood cells, erythrocyte sedimentation rate (ESR), and C-reactive protein (CRP). The preoperative joint aspiration came up positive for *Staphylococcus caprae *(*S. caprae*) infection. Diagnosis and pathogen identification were confirmed by histological examination of six tissue samples obtained during surgery. We initially performed early debridement, antibiotics, and implant retention (DAIR) followed by antibiotic therapy suggested by an infectious disease specialist. DAIR failed two months later, and we proceeded to a two-stage revision. Following surgery, the patient was treated with intravenous antibiotic combination therapy for three weeks and thereafter with oral antibiotics for three months. Four months down the line, the patient is free of symptoms, and the inflammatory markers are normal. Finally, we will proceed with the second stage of revision. This study highlights a very rare case of PJI infection by *S. caprae*, reviews the limited literature, and provides the available evidence for surgical and antibiotic management.

## Introduction

A common pathogen in both community and hospital infections, *Staphylococcus caprae* (*S. caprae*) is a commensal, coagulase-negative Staphylococcus found in the skin flora of goats and humans [1]. In the literature 413 *S. caprae *isolates have been documented globally since the first reported case in 1997 [1], including 55 cases of bone joint infections. It occurs predominantly in the lower limb joints. Twenty-five cases of *S. caprae *infections were examined by Seng et al. [2]. Twenty-four (96%) cases occurred in lower limbs, including nine cases in the knee (36%), four cases in the hip (16%), one case in the femur (4%), four cases in the tibia (16%), three cases in the ankle (12%), and three cases in the foot (12%). Nevertheless, it occurs also in other parts of the body, causing one case of mastoiditis, 27 cases of elbow prosthesis infection, and one case of spondylodiscitis [1,3]. Thirteen patients with periprosthetic joint infection (PJI) were examined by d'Ersu et al., of whom two experienced PJI following total hip arthroplasty (THA) [4]. In summary, including the current case, 10 cases of PJI of the hip have been described in the literature, making them an extremely uncommon condition.

*S. caprae *is capable of creating biofilms on prosthetic materials and adhering to human tissues via fibronectin-binding proteins, successfully evading antibiotics and the body's natural chemotactic leukocyte response [5,6]. *S. caprae *infections have a similar clinical presentation to other Staphylococcus pathogens. Symptoms such as fatigue, pain, swelling, redness, and warmth at the incision site are typical for periprosthetic joint infections. This study highlights a very rare case of PJI infection due to *S. caprae*, reviews the limited literature, and provides the available evidence for surgical and antibiotic management.

## Case presentation

**Figure 1:**
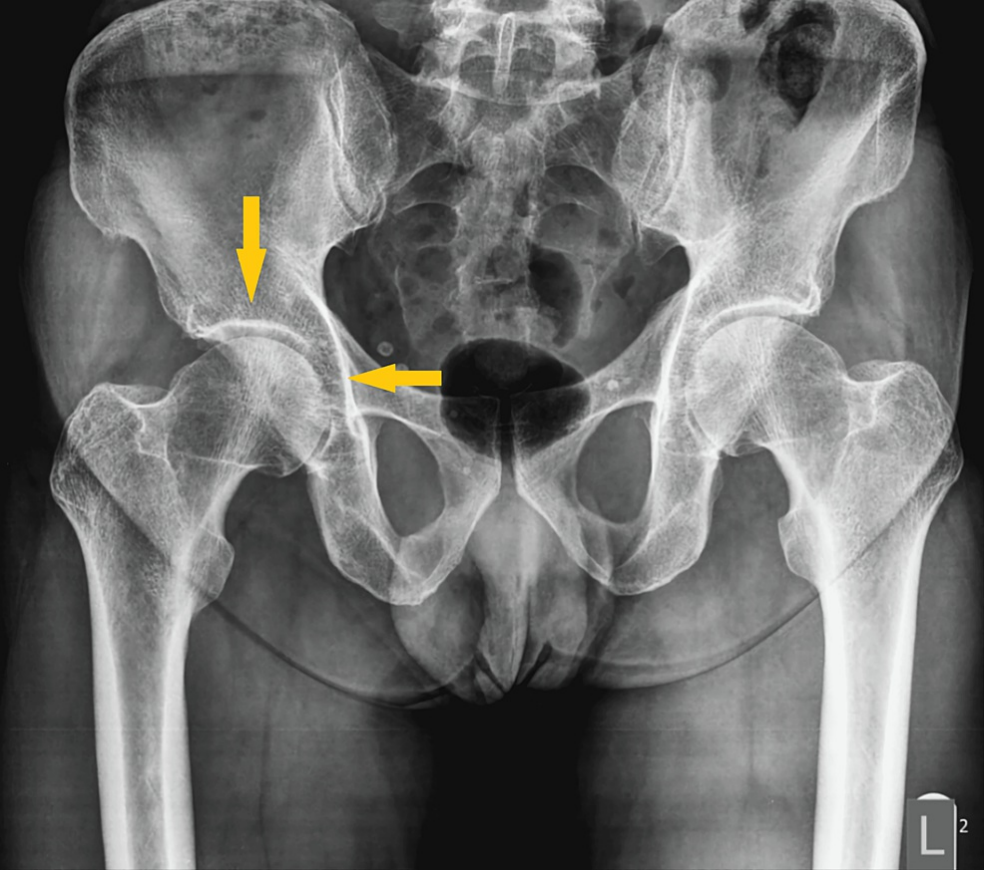
Preoperative anteroposterior plain radiograph of the pelvis. The image shows right hip osteoarthritis. Note joint space narrowing and articular surface sclerosis (arrows).

**Figure 2:**
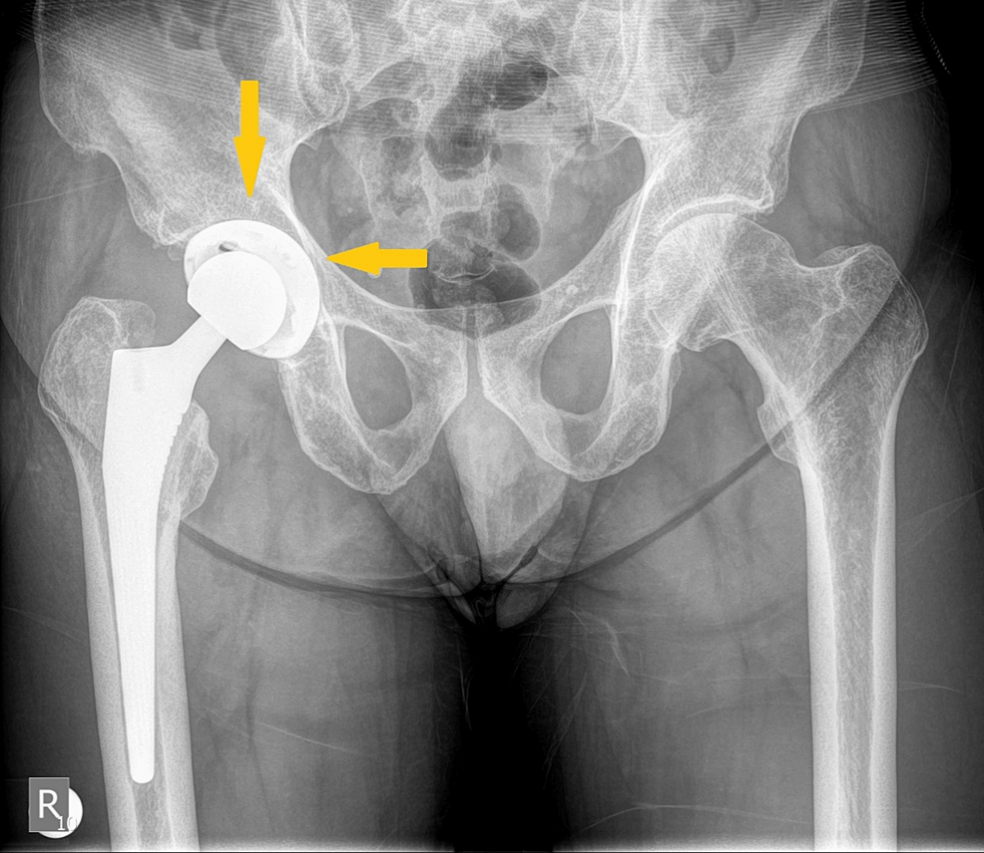
Postoperative anteroposterior plain radiograph of the pelvis. The image shows an uncemented right THA with titanium femoral stem implant and ceramic-on-polyethylene bearing surfaces (arrows). THA: total hip arthroplasty

A 59-year-old male patient underwent a primary uncemented THA in June 2022 due to right hip osteoarthritis (Figure 1). After two days, the patient was discharged without complications (Figure 2).

**Figure 3:**
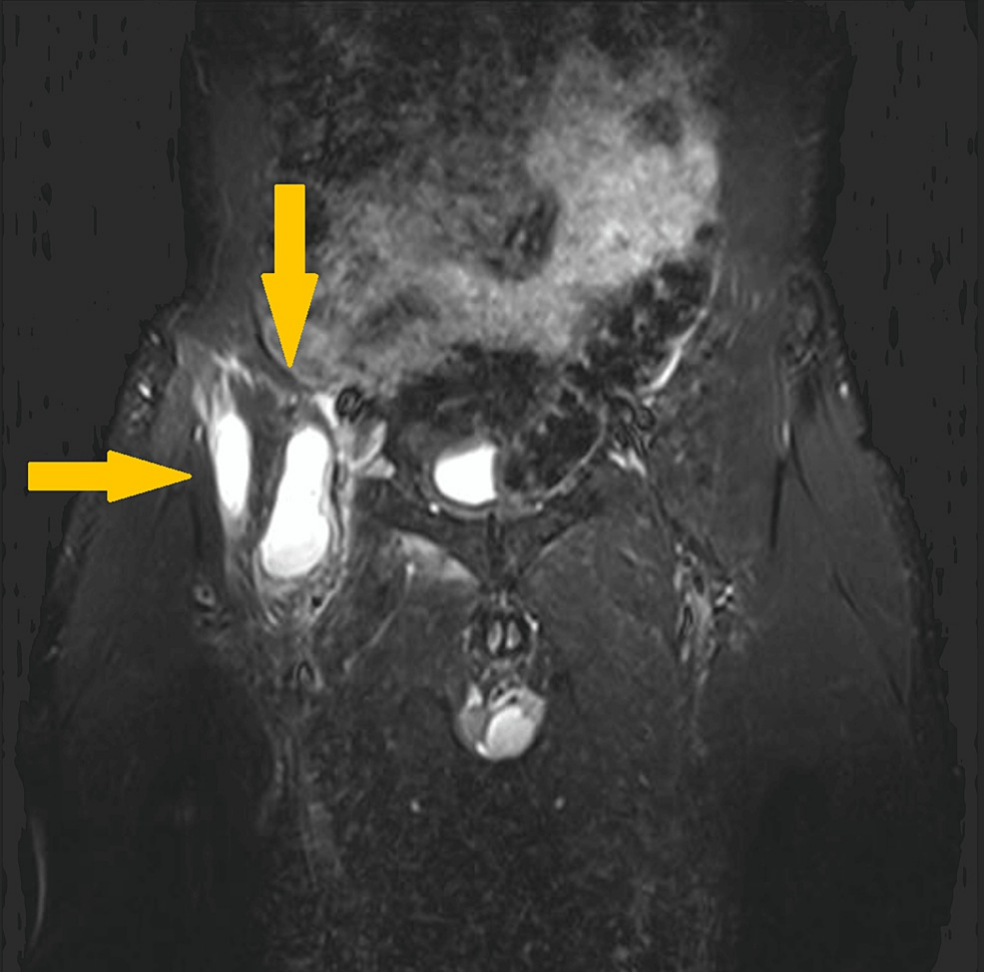
T1-weighted magnetic resonance imaging coronal plane of the pelvis. The image shows a pathologically increased liquid concentration and bone edema around the right hip joint (arrows).

In September 2022, the patient presented febrile (39°C) with groin and buttock pain. The physical examination revealed swelling, redness, and high temperature in the surgical incision area. The results of the laboratory tests showed an ESR of 45 mm and a C-reactive protein (CRP) level of 3.9 mg/dL. The MRI showed a pathologically increased liquid concentration around the right hip joint with bone edema (Figure 3).

Debridement, antibiotics, and implant retention (DAIR) was chosen as the most effective treatment since it was strongly suspected that the prosthetic joint was infected. Synovial fluid was aspirated in the operating room, and six biopsy samples were collected for cultures. All infected tissue was removed by meticulous debridement and irrigation with pulsed lavage, and the removable parts were replaced. Four out of six cultures came positive for *S. caprae*, which was sensitive to most antibiotics. The patient was treated with 1 g intravenous (IV) vancomycin and 400 mg IV ciprofloxacin twice a day for 12 days as an inpatient, followed by 500 mg oral ciprofloxacin and 300 mg oral clindamycin three times a day as an outpatient for three months of antibiotic treatment.

**Figure 4:**
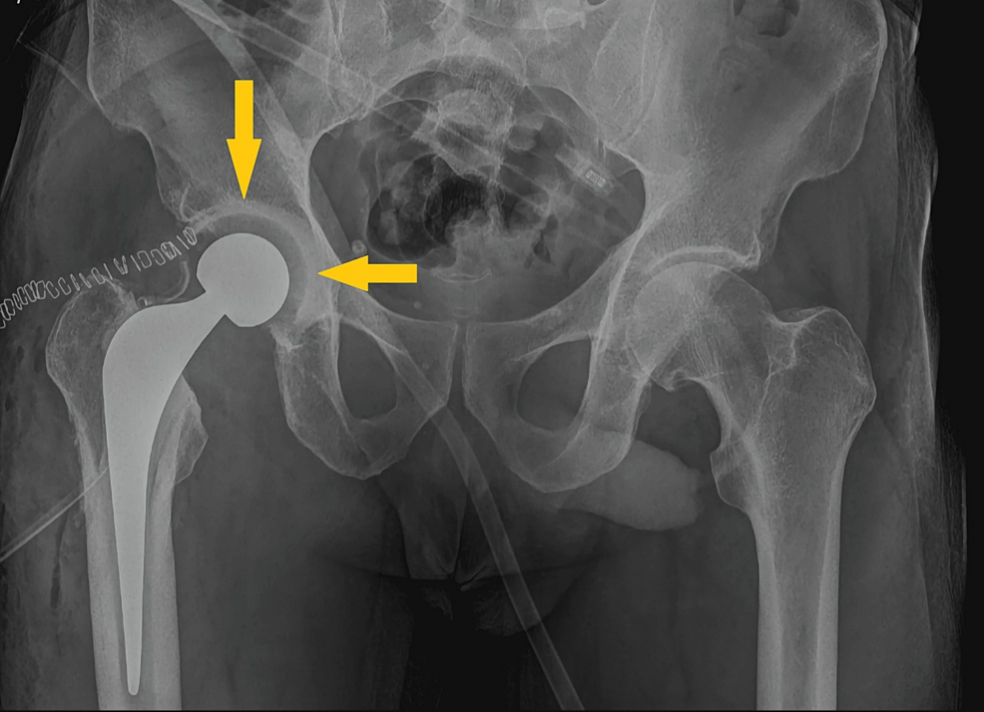
Anteroposterior plain radiograph of the pelvis. The image shows a temporary antibiotic-loaded cement spacer (arrows).

Despite the remission of symptoms and reduced inflammatory markers to normal levels on discharge, the patient was readmitted in December 2022 febrile (38°C) with the same clinical presentation. At the time of admission, the level of CRP was 10.7 mg/dL and ESR 45 mm. This time, a full prosthesis revision was performed. During the operation, synovial and tissue samples were collected for cultures that were negative as expected due to prior antibiotic treatment. The femoral stem and acetabular component were removed, and thorough debridement and irrigation with pulse lavage were performed. Vancomycin-loaded bone cement (1 g per 40 mg of cement) was used, and a fully cemented prosthesis was placed (Figure 4). The patient was hospitalized for 10 days and treated with 1 g IV vancomycin twice a day and 2 g IV cefoxitin three times a day until the inflammatory markers normalized. This was followed by 11 days of outpatient 1 g IV vancomycin and 500 mg IV levofloxacin twice-a-day administration. After completing three weeks of intravenous administration, he continued with 500 mg oral levofloxacin and 600 mg oral rifampin twice daily for three months. Forthcoming, we will proceed to the second-stage revision.

## Discussion

*S. caprae* is a rare pathogen that occurs predominantly in the lower limbs and often affects prosthetic joints, as in this case. There are few reported cases due to the lack of rapid and accurate diagnostic tools and, more likely, a lack of awareness in clinical laboratories and its absence from more commonly used commercially available databases [7,8]. Several diagnostic methods have been used to identify *S. caprae*, such as Vitek, ribotyping 16S-23S printer, soda gene sequencing, and ID32 Staph. Matrix-assisted laser desorption/ionization time-of-flight mass spectrometry (MALDI-TOF MS) is a fast and reliable method, with an identification rate of 97%, for identifying bacteria from agar media [9]. Direct identification from positive blood cultures should decrease the time to obtain the result. In our case, preoperative joint aspiration came up positive for *S. caprae* infection. Diagnosis confirmation was made by cultures examination of the obtained tissue samples.

There is no agreement on the appropriate surgical therapy strategy for patients with *S. caprae *PJIs due to the low incidence. Therapeutic protocols not specific for *S. caprae *are used, including irrigation and debridement with retention of the prosthesis [10], one-stage revision with an exchange of the entire prosthesis (femoral head, femoral stem, acetabular shell, and acetabular liner) [11], two-stage revision with an exchange of the entire prosthesis [12], major partial one-stage revision (removal of either the acetabular shell or femoral stem) [13], or minor partial one-stage revision (where only the femoral head and/or acetabular liner are exchanged) [1]. Seng et al. performed four one-stage and five two-stage revisions out of nine patients [2]. Mencia et al. suggest a one-stage revision with satisfactory results [14]. In our case, we performed DAIR followed by a two-stage revision with a vancomycin-loaded cemented spacer as the first stage. Four months down the line, the patient is free of symptoms, and we will proceed to the second stage.

With an emphasis on patient-reported outcomes for pain and function as well as reinfection rates and cost efficacy, several studies have compared one-stage and two-stage revision [15]. The gold standard was a two-stage revision since it had the lowest incidence of reinfection [16].

Antibiotic treatment in our case included oral levofloxacin and rifampin after IV vancomycin and levofloxacin. Table 1 describes the antibiotic treatment strategies of six articles and 55 cases of *S. caprae *PJIs, including seven hip joints. Overall, 35 cases received two antibiotics, nine received three antibiotics, and nine received one antibiotic. In most cases (43.5%), fluoroquinolone + rifampicin was used, followed by glycopeptide + fluoroquinolone + Rifampicin (10.9%). We suggest a double antibiotic treatment which consists of one fluoroquinolone/glycopeptide/quinolone/penicillin or β-lactam combined with rifampin.

**Table 1:** Antibiotic treatment combinations use (%). ACL: anterior cruciate ligament; PJI: periprosthetic joint infection

Study	Treatment combinations	Antibiotics	Use (%)	Area of infection
Seng et al. [2]	Fluoroquinolone + rifampin	-	18.1	-
Achermann et al. [3]	Fluoroquinolone + rifampin	Ciprofloxacin + rifampin	25.4	Elbow PJI
Darrieutort-Laffite et al. [17]	Fluoroquinolone + rifampin	Ofloxacin + rifampin	1.8	Sacroiliac joint
Mencia et al. [14], Achermann et al. [3], Pommepuy et al. [18]	Quinolone + rifampin	Levofloxacin + rifampin	5.4 (hip), 7.2 (elbow)	Hip PJI, elbow PJI
Achermann et al. [3]	Fluoroquinolone	Ciprofloxacin	1.8	Elbow PJI
Seng et al. [2]	Glycopeptide + fluoroquinolone + rifampin	Vancomycin + rifampin + fluoroquinolone	10.9	-
Seng et al. [2]	Carbapenem + glycopeptide	Vancomycin + imipenem	1.8	-
Seng et al. [2]	Glycopeptide + rifampin + fusidic acid	Teicoplanin + rifampin + fusidic acid	1.8	-
Seng et al. [2]	Glycopeptide	Vancomycin	3.6	-
Achermann et al. [3]	Penicillin + beta-lactamase + rifampin	Amoxicillin- clavulanate + rifampin	1.8	Elbow PJI
Darrieutort-Laffite et al. [17]	Penicillin + rifampin	Oxacillin + rifampin	1.8	Knee PJI
Achermann et al. [3], Elsner et al. [19]	Penicillin	Amoxicillin	3.6 (elbow), 1.8 (knee)	Elbow PJI, knee after an ACL repair
Seng et al. [2]	Sulfonamides + fluoroquinolone + rifampin	Co-trimoxazole + fluoroquinolone + rifampin	1.8	-
Seng et al. [2]	Sulfonamide + fluoroquinolone	Co-trimoxazole + fluoroquinolon	1.8	-
Seng et al. [2]	Sulfonamide + tetracycline	Co-trimoxazole + doxycycline	1.8	-
Seng et al. [2]	Sulfonamides	Co-trimoxazole	1.8	-
Achermann et al. [3]	Fusidic acid + rifampin	Fucidin + rifampin	1.8	Elbow PJI
Seng et al. [2]	Tetracycline	Doxycycline	1.8	-
Achermann et al. [3]	Oxazolidinones	Linezolid	1.8	Elbow PJI

## Conclusions

*S. caprae* is a coagulase-negative Staphylococcus that is extremely uncommon and has a low frequency in the general population. Despite this, it is still an emerging human pathogen in patients with orthopedic infections. There have been only 10 hip PJIs described globally, making them very rare. In the future, identification tools like MALDI-TOF MS, which have demonstrated promising results, will add new data. The therapeutic alternatives are taken from non-*S. caprae*-specific PJI protocols. For a successful clinical outcome, we advise early detection and removal of all implants to control infections embedded in biofilm. Two-stage revision is the gold standard surgical treatment for this type of PJI. For antibiotic treatment, one fluoroquinolone/glycopeptide/quinolone/penicillin or β-lactam should be used in conjunction with rifampicin for better results.
